# Longitudinal Intravital Imaging of the Retina Reveals Long-term Dynamics of Immune Infiltration and Its Effects on the Glial Network in Experimental Autoimmune Uveoretinitis, without Evident Signs of Neuronal Dysfunction in the Ganglion Cell Layer

**DOI:** 10.3389/fimmu.2016.00642

**Published:** 2016-12-23

**Authors:** Daniel Bremer, Florence Pache, Robert Günther, Jürgen Hornow, Volker Andresen, Ruth Leben, Ronja Mothes, Hanna Zimmermann, Alexander U. Brandt, Friedemann Paul, Anja E. Hauser, Helena Radbruch, Raluca Niesner

**Affiliations:** ^1^German Rheumatism Research Center, Berlin, Germany; ^2^NeuroCure Clinical Research Center, Clinical and Experimental Multiple Sclerosis Research Center, Department of Neurology, Charité – Universitätsmedizin Berlin, Berlin, Germany; ^3^Luigs & Neumann GmbH, Ratingen, Germany; ^4^LaVision Biotec GmbH, Bielefeld, Germany; ^5^Department of Neuropathology, Charité – Universitätsmedizin, Berlin, Germany; ^6^Immundynamics, Charité – Universitätsmedizin Berlin, Berlin, Germany

**Keywords:** longitudinal retina imaging, experimental autoimmune uveoretinitis, neuronal calcium imaging, chronic inflammation, multiphoton microscopy

## Abstract

A hallmark of autoimmune retinal inflammation is the infiltration of the retina with cells of the innate and adaptive immune system, leading to detachment of the retinal layers and even to complete loss of the retinal photoreceptor layer. As the only optical system in the organism, the eye enables non-invasive longitudinal imaging studies of these local autoimmune processes and of their effects on the target tissue. Moreover, as a window to the central nervous system (CNS), the eye also reflects general neuroinflammatory processes taking place at various sites within the CNS. Histological studies in murine neuroinflammatory models, such as experimental autoimmune uveoretinitis (EAU) and experimental autoimmune encephalomyelitis, indicate that immune infiltration is initialized by effector CD4^+^ T cells, with the innate compartment (neutrophils, macrophages, and monocytes) contributing crucially to tissue degeneration that occurs at later phases of the disease. However, how the immune attack is orchestrated by various immune cell subsets in the retina and how the latter interact with the target tissue under *in vivo* conditions is still poorly understood. Our study addresses this gap with a novel approach for intravital two-photon microscopy, which enabled us to repeatedly track CD4^+^ T cells and LysM phagocytes during the entire course of EAU and to identify a specific radial infiltration pattern of these cells within the inflamed retina, starting from the optic nerve head. In contrast, highly motile ${\text{CX}}_{\text{3}}{\text{CR}}_{1}^{+}$ cells display an opposite radial motility pattern, toward the optic nerve head. These inflammatory processes induce modifications of the microglial network toward an activated morphology, especially around the optic nerve head and main retinal blood vessels, but do not affect the neurons within the ganglion cell layer. Thanks to the new technology, non-invasive correlation of clinical scores of CNS-related pathologies with immune infiltrate behavior and subsequent tissue dysfunction is now possible. Hence, the new approach paves the way for deeper insights into the pathology of neuroinflammatory processes on a cellular basis, over the entire disease course.

## Introduction

Autoimmunity against compartments of the central nervous system (CNS) can lead to the development of chronic neuroinflammatory diseases, of which multiple sclerosis (MS) is the main representative. Frequently, the very first clinical signs of MS disease course are related to transient loss of vision (1–3). In the case of neuromyelitis optica (NMO), another chronic neuroinflammatory disease with strong involvement of the retina, permanent damage of visual function represents a clinical hallmark (4–6). Thinning of the neuronal retina, i.e., retinal neural fiber layer and the ganglion cell layer (GCL), has been demonstrated using optical coherence tomography (OCT) both in MS and NMO patients (7, 8). Whether this loss of neuronal tissue in the retina is caused by immune infiltration at distal parts of the optic nerve or by a direct immune attack on neuronal retina components remains unclear.

Although experimental autoimmune uveoretinitis (EAU) is not a classical model of neuroinflammation, it mimics CNS autoimmunity within the retina and resembles complementary features of chronic neuroinflammation as compared to experimental autoimmune encephalomyelitis (EAE). Various EAU models in mouse and rat have been developed to highlight the contribution of different immune cell subtypes to autoimmunity in the eye (9). In this study, we use an EAU model with immunization of C57/B6-J mice against the first peptide sequence (1–20) of the interphotoreceptor retinoid-binding protein (IRBP). The latter protein is found in the extracellular space between photoreceptors and the retinal pigment epithelium (RPE). Previous histological studies have shown that in this model autoreactive CD4^+^ T cells pass the blood–retina barrier, infiltrate the retina, and attract cell subsets of the innate immune system from the periphery and from within the CNS. Presumably, visual loss is caused by retinal tissue degeneration, especially detachment of the photoreceptor layer. This hypothesis is supported by fundoscopy, histology, OCT, and electroretinography results of other studies (10, 11). However, the dynamics of the immune infiltrate and its effects on the function of the neuronal retina, as the central tissue responsible for intact visual function, have not yet been investigated, due to the lack of suitable technologies. In this context, technologies—such as the longitudinal intravital two-photon imaging—are needed, which open the possibility for non-invasive, dynamic 3D image acquisition in the mouse retina.

While two-photon microscopy of retinal whole mounts has already been established and applied to elucidate central questions in neurosciences and neurobiology (12–14), intravital retinal imaging in the mouse has only recently been demonstrated as viable in the context of ophthalmologic diseases (15–17). In studies using the technology, either adaptive optics (16) or a periscope-based setup (18) was used to visualize changes to the RPE in various mouse models. However, the complexity of the optical setup limited the imaging results to single static images.

In this work, we describe a new approach for intravital retinal two-photon imaging and demonstrate its advantages in the context of chronic inflammation in the eye. The main benefit of the approach as compared to established techniques such as OCT, electroretinography (19–21), or scanning laser ophthalmoscopy is its ability to assess cellular motility patterns as well as tissue function and dysfunction over the whole disease course, at cellular and subcellular resolution. Additionally, our setup does not require adaptive or other complex optics, thus, allowing for easy and intuitive use. Employing our intravital retinal imaging approach, we quantified the specific motility pattern of CD4^+^, LysM^+^, and ${\text{CX}}_{\text{3}}{\text{CR}}_{1}^{+}$ cells in the inflamed retina and observed the morphologic shift within the glial network induced by the immune infiltrate in EAU. No sustained neuronal dysfunction of the ganglion cells associated with sustained increased cellular calcium was detected during the first phase of EAU, in contrast to the results found under similar inflammatory conditions in the brain (22). This finding indicates that the intracellular calcium signaling in the neuronal retina is not primarily affected by the inflammation in our EAU disease model. Although intracellular calcium is generally accepted to be associated with neuronal function and dysfunction, we do not exclude that neuronal retinal dysfunction may appear later during disease course.

## Results

### Clinical Scoring and Characterization of Cellular Markers during EAU

We employed fundoscopy in C57/Bl6-J mice immunized with IRBP (peptide 1–20) to resume previous findings regarding the clinical symptoms of EAU at the onset and peak of the disease, up to day 28 after immunization and, thus, to validate our experimental setup (Table 1). We observed perivascular cellular accumulations (cuffing), changes in the optic disc aspect due to immune infiltration and widening of the main retinal blood vessels (Figure S1A in Supplementary Material), which are previously described hallmarks of the disease (10). Clinical scoring considering these criteria—summarized in Table 2—shows an exacerbation of the disease over time (*n* = 10 mice, three independent EAU experiments, Figure S1B in Supplementary Material).

**Table 1:** **Mouse strains and experimental autoimmune uveoretinitis (EAU) experiment code for all mice included in longitudinal multiphoton imaging of the retina**.

Mouse strain	Experiment ID
CerTN L15 × LysM tdRFP	EAU-1
CerTN L15 × LysM tdRFP	EAU-2
CerTN L15 × LysM tdRFP	EAU-2
LysM tdRFP	EAU-5
LysM tdRFP	EAU-5
LysM tdRFP	EAU-5
CerTN L15 × LysM tdRFP	Healthy
CerTN L15 × LysM tdRFP	Healthy
CerTN L15 × LysM tdRFP	Healthy
CerTN L15 × LysM tdRFP	Healthy
${\text{CX}}_{3}{\text{CR}}_{1}^{+/-}$ EGFP	EAU-4
${\text{CX}}_{3}{\text{CR}}_{1}^{+/-}$ EGFP	EAU-4
${\text{CX}}_{3}{\text{CR}}_{1}^{+/-}$ EGFP	EAU-4
${\text{CX}}_{3}{\text{CR}}_{1}^{+/-}$ EGFP	Healthy
${\text{CX}}_{3}{\text{CR}}_{1}^{+/-}$ EGFP	Healthy
CD4^+^ eYFP	EAU-3
CD4^+^ eYFP	EAU-3
CD4^+^ eYFP	EAU-3
CD4^+^ eYFP	Healthy
CD4^+^ eYFP	Healthy

*The mice were also scored by fundoscopy and analyzed at day 28 after immunization using histology and immunofluorescence techniques*.

**Table 2:** **Experimental autoimmune uveoretinitis grading criteria in mice with C57BL/6 background adapted from Xu et al. (23)**.

Score	Optic disc inflammation	Retinal vessels	Retinal tissue
1	Minimal	Engorged vessels	1–4 small lesions
2	Mild	Engorged vessels and 1–4 mild cuffings	>4 small lesions or 2–3 linear lesions
3	Moderate	>4 mild cuffings or 1–3 moderate cuffings	>4 small lesions and >3 linear lesions
4	Severe	>3 moderate cuffings or severe cuffings	Linear lesion confluent

In line with previous reports (9, 10, 19), immunofluorescence and histology (HE) analysis of the retina at peak of EAU corroborated our fundoscopy results. As highlighted by DAPI and HE staining in cross sections of the eye, the highly ordered retinal layer structure common to healthy controls becomes wave-shaped (Figure S1C in Supplementary Material) and massive immune cell infiltration was found throughout all retinal layers and in the vitreous humor in the EAU mice, but not in healthy controls.

Immunofluorescence analysis showed an increase of Iba1^+^ cell number, indicating microglial activation. Similarly, the glial fibrillary acidic protein (GFAP) signal (astrocytes) within the GCL increased, indicating astrocytic activation as previously found in the inflamed brain (24) (Figure S1D in Supplementary Material).

### Setup for Longitudinal Intravital Multiphoton Imaging of the Mouse Retina

In this study, we present a novel *in vivo* microscopy setup that facilitates the study of immune system dynamics in the retina. The new technology, based on time-lapse multiphoton fluorescence imaging of the retina at subcellular resolution, allows not only quantification of immune infiltration but also monitoring of tissue dysfunction.

**Figure 1:**
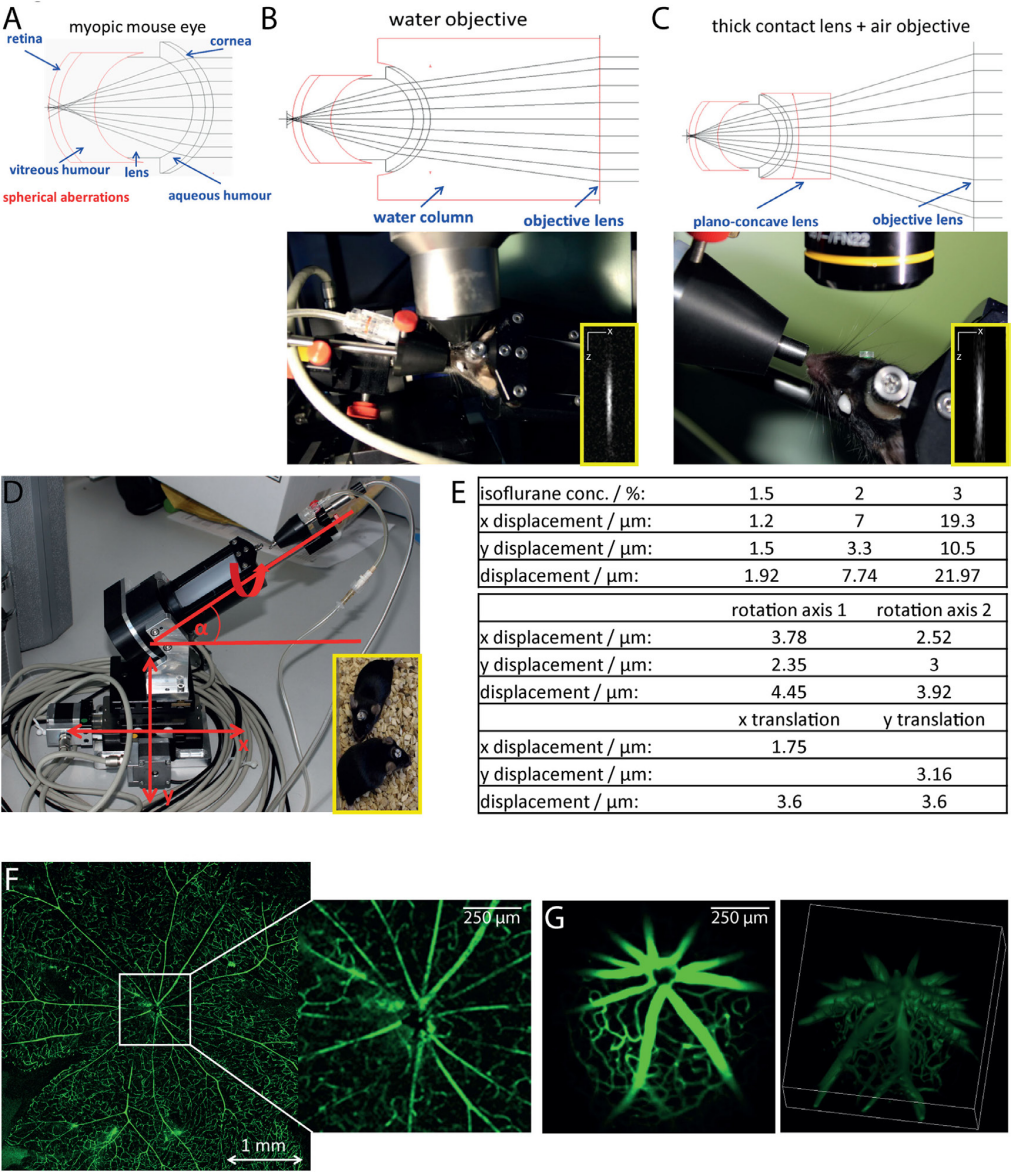
**Longitudinal intravital retinal imaging setup for monitoring cellular dynamics and functions over time**. **(A)** Simulation of the myopic mouse retina shows that spherical aberration hampers focusing on the mouse retina. **(B)** A simple and robust solution, as demonstrated by the simulation in the upper image, is the use of water-immersion objectives with large working distances (touchless imaging). The objective lens used is a 4× magnification water-immersion objective lens, NA = 0.28, and WD 6 mm, as shown during imaging in the lower image. Lateral resolution: 1 μm, axial resolution 11 μm at excitation wavelength 850 nm. **(C)** Simulations of the optical path (upper image) show that using corresponding plan-concave lenses together with an air objective lens also allow focusing onto the mouse retina. We placed a plan-concave lens of 3 mm diameter using hydrogel on the eye of the mouse and focused the excitation beam using a 10× air objective NA = 0.3 and WD 18 mm. The insets show *x* and *z* projections of the point spread function of the system measured on 100 nm fluorescent beads (emission 605 nm), at 850 nm excitation wavelength. **(D)** The mouse positioning system allows translation on three different axis (*x, y*, and *z*) and rotation around two axes with a mechanical reproducibility of 2 μm. The fixation on the positioning system is based on a titanium alloy head-post mounted on the skull of the mouse 2 weeks before starting the experiments [inset of **(D)**]. **(E)** The tables overview the positioning reproducibility of the setup in **(D)** during 100 repeated acquisition steps of the vasculature labeled with FITC dextrane, when varying the anesthesia depth given by the isoflurane concentration (upper table) and shifting the rotational axis (middle table) and the translational axis (*x* and *y*, lower table). Comparison of image quality of the retina vasculature (FITC dextrane) as visualized in flat mounts **(F)** and by longitudinal intravital microscopy **(G)**. Whereas the imaging quality is similar, the two-photon microscopy allows for 3D visualization of the central region of 1 mm × 1 mm in the retina.

Since the eye of the mouse is highly myopic, focusing the excitation laser beam of a multiphoton microscope on the retina is challenging due to spherical aberrations, especially due to third order spherical aberration (25). The mouse eye was modeled as an optical multilens system comprising cornea, aqueous body, lens, vitreous humor, and retina, taking into account typical refractive indexes of the single components and their curvature [Figure 1A; (25)]. We found that the spherical aberrations in the myopic mouse eye can be corrected if focusing the excitation beam through a water-immersion objective lens, thus allowing for high-quality imaging of the retina (Figure 1B). The use of air objective lenses to image the retina is only possible if using an additional contact lens (in our case, a plano-concave lens, diameter: 3 mm, center thickness: 1 mm, radius of curvature: −1.6 mm, TT Optics GmbH), to correct the spherical aberrations (Figure 1C).

In order to avoid damage of the cornea due to dehydration during imaging, we decided to use a water-immersion objective lens in our microscopy setup. The challenge in this respect is the necessity for large working distances of the objective lenses (>4 mm), as the average diameter of the mouse eye at 10 weeks of age is approximately 3.2 mm (25). Additionally, to ensure non-invasive repeated imaging of the same mouse, we avoided direct mechanical contact between cornea and objective lens and limited the image acquisition time to 20 min.

We designed a water-immersion objective lens with a working distance of 6 mm and an effective NA of 0.28 (Figure 1B), using nearly the aperture of the mouse eye (0.32) made possible by pharmaceutically widening the pupil (mixture of 2% phenylephrine and 0.4% tropicamide) directly before starting the imaging experiments. The spatial resolution of the 10× air objective (NA = 0.3) was 1.25 μm laterally and 14.1 μm axially at 850 nm excitation wavelength, which allowed visualization of three-dimensional cellular details. Similarly, the resolution of the 4× water-immersion objective (NA = 0.28) was 1.33 μm laterally and 16.3 μm axially after excitation at 850 nm.

In order to find the orientation of the eye during repeated imaging sessions, we developed a positioning stage with two translational and two rotational axes (Figure 1D). The mouse was fixed onto this stage using a biocompatible titanium alloy head-post mounted onto the skull 2 weeks prior to performing experiments (Figure 1D, inset). The head-post was glued to the skull using dental cement, which ensures high stability of the fixation and does not have any side effects on the mouse. The positioning system additionally contained an inhalation mask, which enables highly accurate control of the anesthesia and, hence, of the eye movement. The mechanical reproducibility of the positioning system using our system was 2 ± 1.5 μm for the translation axes (*x* and *y*). The repeated eye positioning under *in vivo* conditions was strongly influenced by the level of animal anesthesia (Figure 1E), but does not exceed 10 μm under optimal anesthesia conditions (Figures 1E, *N* = 100 repositioning steps). The displacement can be easily corrected using the vasculature of the eye as reference system. In summary, thanks to the positioning system, data were generated at the same imaging position during repeated imaging sessions. This allowed us to compare the data sets acquired at various time points from the same animal. Repeated intravital imaging experiments in healthy CD4^+^.eYFP, ${\text{CX}}_{3}{\text{CR}}_{1}^{+/-}$ EGFP, and CerTN L15 × LysM tdRFP mice—summarized in Table 1—using our setup showed no evident signs of either retinal inflammation or calcium-associated neuronal retinal dysfunction, as is often the case of repeated anesthesia, surgery, or mechanical stress during the imaging process (Figure S2 in Supplementary Material).

In order to verify the optical performance of our device, we compared the image of a retinal flat mount with the two-photon image of the retina in a living anesthetized mouse after injection with FITC dextrane for labeling of the vasculature (Figures 1F,G). Both imaging methods were able to resolve even fine capillaries with high accuracy, while only the multiphoton imaging restored the curvature of the retina by 3D imaging (Video S1 in Supplementary Material). The effective field of view under *in vivo* conditions reached 1.5 mm × 1.5 mm, which was, as expected, smaller than that of the flat mount.

The key advantage of the multiphoton microscopy is the ability to longitudinally image the retina in living animals, i.e., over weeks, so that the entire disease course can be investigated in a single animal. We also employed our technology to gain novel insight into the pathologic processes during EAU as a model of chronic inflammation in the eye.

### Dynamics of Retinal Infiltration with Peripheral Immune Cells in EAU

**Figure 2:**
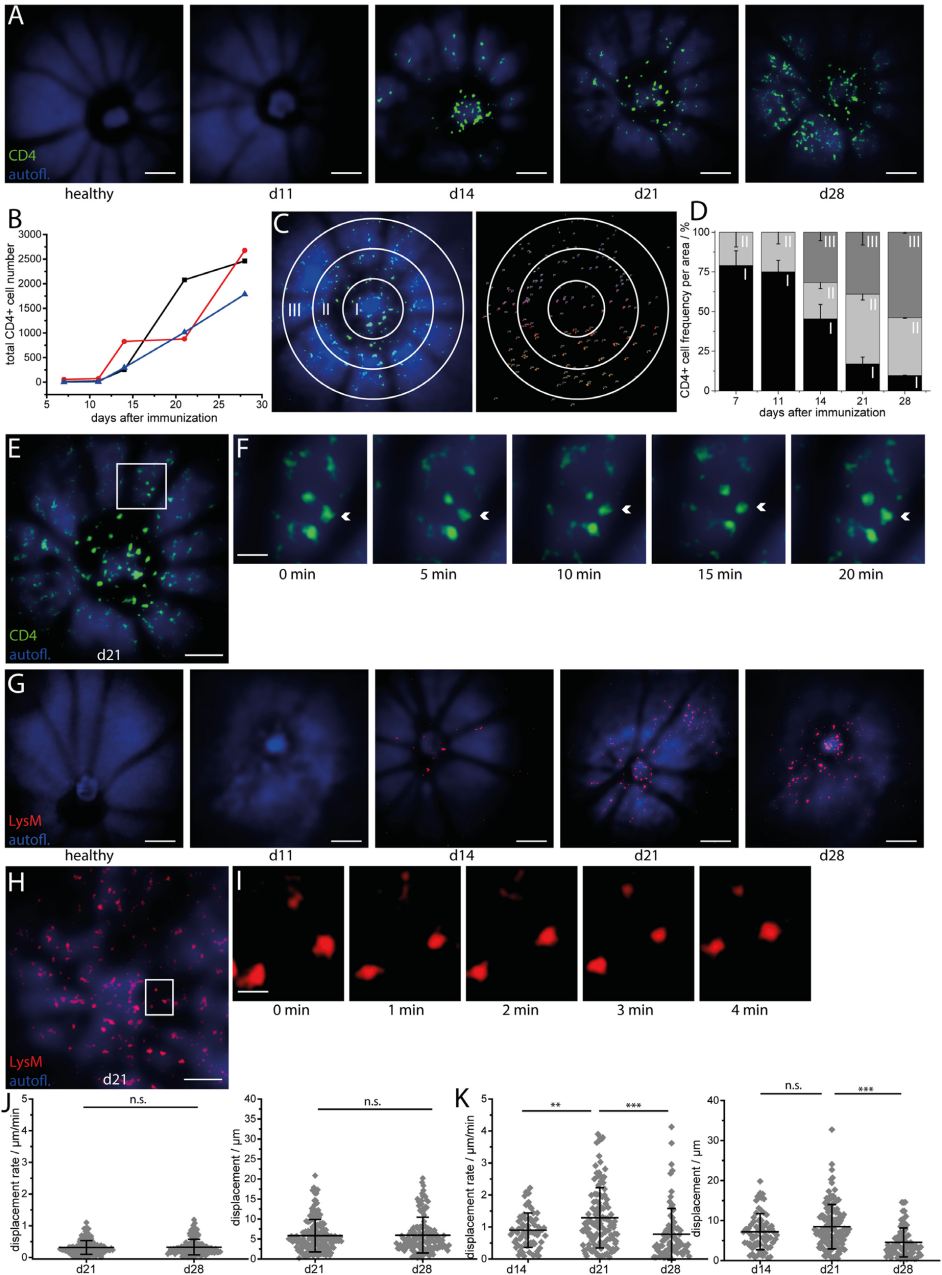
**Longitudinal intravital imaging pinpoints CD4^+^ T cells and LysM^+^ phagocytes infiltration of the retina as a slow but highly oriented process during experimental autoimmune uveoretinitis (EAU)**. **(A)** Representative three-dimensional projections of fluorescence retinal images (1,400 μm × 1,400 μm × 300 μm) in a healthy mouse and a EAU mouse at days 11 (pre-onset), 14 (onset), 21 (peak), and 28 (peak) during the disease course. Autofluorescence mainly originating from retinol in the retinal pigment epithelium (RPE) is depicted in blue and serves for orientation, since blood vessels and the optic nerve head do not fluoresce and appear as shadows. CD4^+^ eYFP cells are depicted in green and first appear within the retina at EAU onset (day 14 after immunization), starting from the optic nerve head. λ_exc_ = 960 nm, λ_detection_ = 593 ± 20 nm (eYFP), and 525 ± 25 (autofl. retinol). Scale bar = 200 μm. **(B)** Increase of the total number of CD4 eYFP cells in the single mice affected by EAU during the course of the disease (*n* = 3 EAU mice; *n* = 2 healthy mice). **(C)** Representative 3D projection of the retina infiltrated by CD4 eYFP cells at day 21 after immunization and object recognition in the same image, following fluorescence intensity and object size criteria. The white circles define three concentric regions around the optic nerve head (Region I: 0—226 μm; Region II: 226—452 μm; Region III: 452—678 μm from the center of the optic nerve head), in which the cells were counted to quantify the distribution of the CD4^+^ population in EAU mice. **(D)** Spatial distribution of CD4 eYFP cells within the concentric regions defined in **(C)** during the course of EAU. The infiltration of the retina with CD4^+^ T cells is a slow but highly oriented process, starting from the optic nerve and spreading toward the retinal edge. Overview retinal fluorescence image of CD4 eYFP cells at day 21 after immunization **(E)** and 20 min time-lapse images of the white square inset **(F)** show that the cells are mostly sessile (Videos S2 and S3 in Supplementary Material). Acquisition rate = 1,400 μm × 1,400 μm × 300 μm *z*-tack/min. Scale bar **(E)** = 200 μm. Scale bar **(F)** = 50 μm. **(G)** Representative three-dimensional projections of fluorescence retinal images (1,400 μm × 1,400 μm × 300 μm) in a healthy mouse and in a mouse affected by EAU at time point days 11 (pre-onset), 14 (onset), 21 (peak), and 28 (peak) during the course of the disease. Autofluorescence mainly originating from retinol within the RPE is depicted in blue and serves for orientation, since blood vessels and the optic nerve head do not fluoresce and appear as shadows. LysM tdRFP cells are depicted in red and first appear within the retina at EAU onset (day 14 after immunization), starting from the optic nerve head and from the main vessels. λ_exc_ = 1,100 nm, λ_detection_ = 593 ± 20 nm (tdRFP), and 525 ± 25 (autofl. retinol). Scale bar = 200 μm. **(H)** 3D projection of fluorescence images of LysM + tdRFP cells (red) infiltrating the retina (retinol autofluorescence, blue) at day 21 after immunization. **(I)** Five minutes time-lapse image of the white square inset in **(H)** show that LysM^+^ cells are also moving only slowly (Videos S4 and S5 in Supplementary Material). Acquisition rate = 1,400 μm × 1,400 μm × 200 μm *z*-tack/min. Scale bar **(H)** = 200 μm. Scale bar **(I)** = 50 μm. **(J)** Displacement and displacement rate of CD4^+^ T cells within the retina at days 21 and 28 after immunization (*n* = 4 EAU mice, *n* = 4 healthy mice). **(K)** Displacement and displacement rate of LysM tdRFP phagocytes within the retina at days 14, 21, and 28 after immunization. Statistical evaluation was determined by ANOVA one-way tests and Bonferroni *post hoc* test for multicolumn analysis (*p < 0.05, **p < 0.01, and ***p < 0.001).

We performed repeated intravital multiphoton imaging of CD4.eYFP mice during the course of EAU at 7, 11, 14, 21, and 28 days after immunization with IRBP_1–20_. In line with previous immunofluorescence data and with our fundoscopic clinical score, we found an increase in CD4^+^ T cell number over the course of EAU in all investigated mice (Figures 2A,B; *n* = 3 mice, Table 1) beginning from day 14 (onset of disease). Longitudinal intravital imaging revealed that CD4^+^ T cells infiltrated the retina starting from the optic disc and diffused radially. Analysis of the CD4^+^ cell infiltration in a concentric zonal sectioning of the retina (area I 430 μm diameter, centered the middle of the optic disc, area II 430 to 860 μm diameter, and area III 860 to 1,250 μm diameter) showed that the immune infiltration increases in the peripheral areas and decreases at the optic nerve head during EAU (Figures 2C,D). Interestingly, most of CD4^+^ T cells in the retina are not showing strongly directed displacement during the imaging window of 20 min (Figures 2E,F,J); Videos S2 and S3 in Supplementary Material), neither at day 21 nor at day 28 after immunization. At day 14 after immunization, the CD4^+^ cells were clustered at the optic nerve head and no trajectories could be recorded. The global displacement vector of CD4^+^ cells at all investigated time points during EAU, describing either centrifugal or centripetal motion, showed no preferential short-term motion of these cells. The mean displacement rate of CD4^+^ cells infiltrating the retina was reduced as compared to that of CD4^+^ T cells in the brain stem, during EAE (22). These findings imply that the CD4^+^ T cell infiltration of the retina, although a highly directed overall process, is rather slow.

Repeated intravital imaging experiments in the retina of LysM tdRFP mice affected by EAU (*n* = 4 mice, Table 1, Figures 2G–I, Videos S4 and S5 in Supplementary Material) revealed that LysM^+^ cells (peripheral phagocytes—macrophages, monocytes, and neutrophils) behave similar to CD4^+^ cells. The LysM^+^ cells enter the retina at the optic nerve head and the main retinal blood vessels at day 14 after immunization (Figure 2G). The time point of their entry was slightly later than that of CD4^+^ cells, as proportionally less LysM^+^ cells than CD4^+^ T cells were found at day 14 after immunization as compared to later time points (days 21 and 28). This observation lends weight to the hypothesis that CD4^+^ T cells initiate the retinal inflammation in EAU and attract cells of the innate immune system to the eye, e.g., LysM^+^ cells.

The displacement rates of LysM^+^ cells were slower than the displacement rates of LysM^+^ cells in the brain stem of EAE mice, but significantly higher than that of CD4^+^ T cells in the retina during EAU (***p < 0.001, ANOVA statistical evaluation). Interestingly, both the displacement and the displacement rate of LysM^+^ cells were significantly higher at day 21 than days 28 and 14 (onset EAU) (Figure 2K). The global displacement vector for highly motile LysM^+^ cells in EAU, at days 14 and 21, amounted to 117.1 and 146.8 μm, respectively. This indicates a preferential centrifugal motion, away from the optic nerve. At day 28, the LysM^+^ cells showed a slight centripetal motion (global displacement vector −34.6 μm).

The typical second harmonics generation signal of highly ordered collagen fibers were not detectable (Figures 2B,G) at any point during the EAU course. The collagen fibers typically build the conduits in secondary lymphoid organs and serve as highways for immune cells in the inflamed brain.

### Retinal CX_3_CR_1_ Subset Shifts toward an Activated Morphology and Exhibits Centripetal Motion during EAU

**Figure 3:**
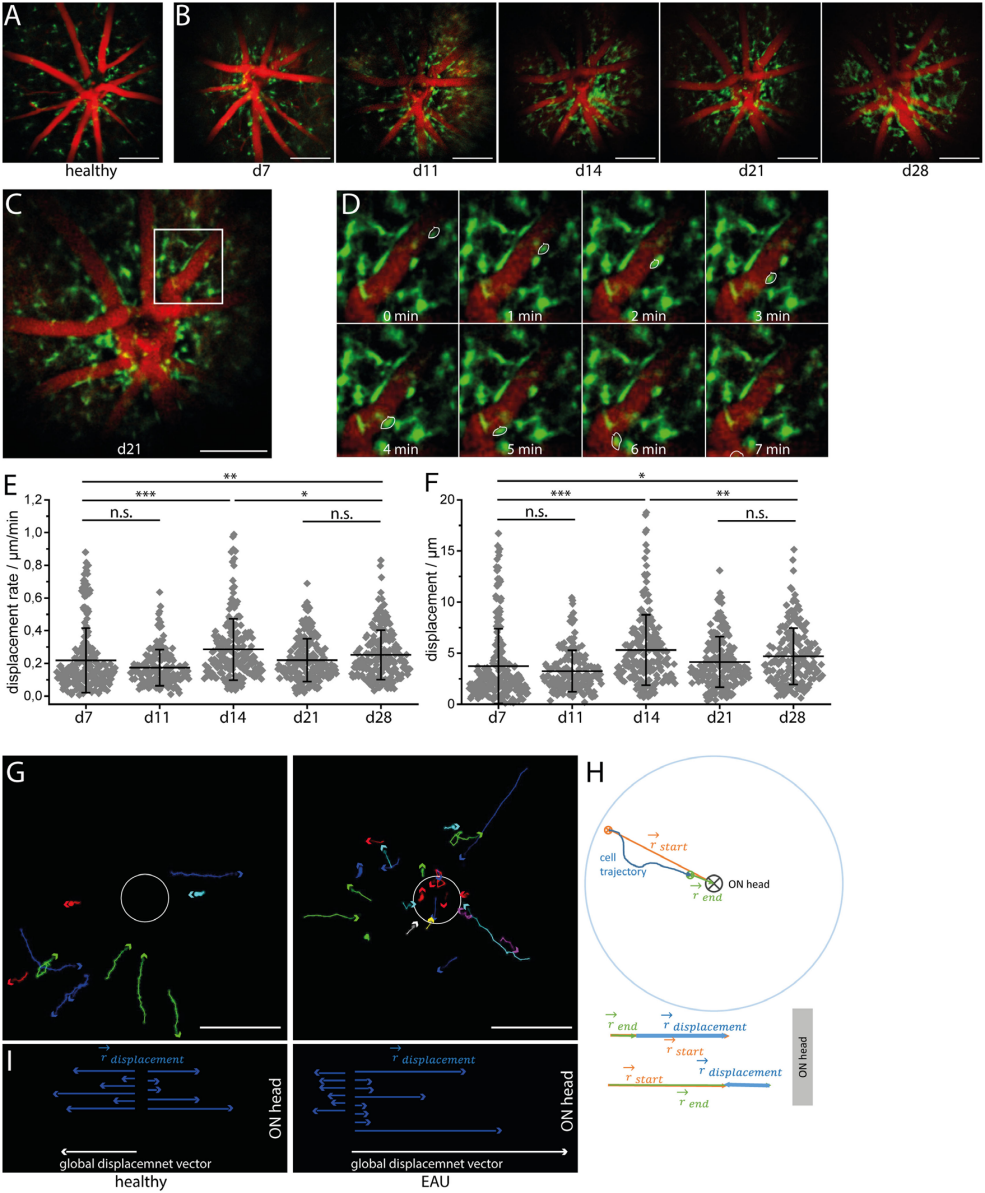
**CX_3_CR_1_ cell subset shifts toward an activated morphology and shows preferential centripetal motion during experimental autoimmune uveoretinitis (EAU)**. Sample three-dimensional projections of fluorescence retinal images (1,400 μm × 1,400 μm × 300 μm) in a healthy mouse **(A)** and in a ${\text{CX}}_{3}{\text{CR}}_{1}^{+/-}$eGFP mouse affected by EAU at time points days 7 (pre-onset), 11 (pre-onset), 14 (onset), 21 (peak), and 28 (peak) during the course of the disease **(B)**. Blood vessels are labeled by rhodamine (red) and ${\text{CX}}_{3}{\text{CR}}_{1}^{+/-}$ eGFP microglia/macrophages are shown in green. During EAU, some CX_3_CR_1_ cells shifted from the typical resting microglial morphology toward an amoeboid morphology, which is typical for the phagocytic phenotype. λ_exc_ = 850 nm, λ_detection_ = 525 ± 25 nm (eGFP), and 593 ± 20 (rhodamine). *z*-step = 15 μm. Scale bar = 200 μm. Overview **(C)** and corresponding time-lapse of 3D projections of fluorescence images of CX_3_CR_1_ eGFP cells and of the vasculature in white square inset within the retina **(D)** over 20 min. Acquisition rate = 1,400 μm × 1,400 μm × 300 μm/min. *z*-step = 15 μm. The depicted CX_3_CR_1_ cell (white outline) is moving within the perivascular space with a speed of 31 μm/min. **(E)** Displacement rate and displacement of eGFP^+^ cells during the course of EAU at days 7, 11, 14, 21, and 28 after immunization (*n* = 3 EAU mice, *n* = 2 healthy controls). **(F)** Tracks of cells showing a displacement larger than 20 μm over 20 min in healthy mice and mice affected by EAU. Scale bar = 200 μm. **(G)** Displacement vectors of single motile cells toward/away from the optic nerve head and the global displacement vector for each situation: health and EAU. **(H)** Principle of assessing the displacement vector toward/away from the optic nerve head for single cells. Statistical evaluation was determined by ANOVA one-way tests and Bonferroni *post hoc* test for multicolumn analysis (*p < 0.05, **p < 0.01, and ***p < 0.001).

Longitudinal intravital multiphoton imaging of the retina in CX_3_CR_1_.EGFP mice affected by EAU revealed a change in the behavior of ${\text{CX}}_{3}{\text{CR}}_{1}^{+}$ cells from a probing toward a phagocytic morphology. In healthy controls as well as in mice at days 7 and 11 after immunization (pre-onset), most of the ${\text{CX}}_{3}{\text{CR}}_{1}^{+}$ cells were microglia and had a highly ramified morphology. In contrast, amoeboid cells (phagocytes, mainly activated microglia, and macrophages) appeared, particularly around the vessels and at the optic nerve head, starting from day 14 after immunization, i.e., EAU onset (Figures 3A,B) but were practically absent in the parenchyma. Time-lapse imaging after immunization revealed a leaky retinal vasculature in EAU mice, but not in healthy controls, which indicates disruption of the blood–retina barrier (Videos S6–S9 in Supplementary Material). Most ${\text{CX}}_{3}{\text{CR}}_{1}^{+/-}$ EGFP cells remained mostly sessile, showing low displacement and reduced displacement rate (Figures 3C–F). The values of both displacement and displacement rate of the cells reached a maximum at day 14 after immunization (EAU onset) and were decreased at later time points (days 21 and 28 after immunization, EAU peak). During onset and peak EAU, i.e., in mice having clinical symptoms, these values were significantly higher than during the pre-onset phase (days 7 and 11 after immunization), i.e., in mice showing no clinical signs. The small population of ${\text{CX}}_{3}{\text{CR}}_{1}^{+}$ cells showing significant displacement (>10 μm) moved exclusively in the perivascular space of main retinal vessels (Figure 3D; Videos S6–S9 in Supplementary Material) in both healthy controls and mice affected by EAU. Their mean displacement rate (9.55 ± 2.63 μm/min in healthy controls and 10.55 ± 4.80 μm/min mice affected by EAU) was similar to that measured for CD4^+^ cells moving within the perivascular space, in the brain stem of mice affected by EAE (26). The displacement vectors of all motile cells were summed up in healthy and EAU-affected mice, respectively, to determine the global displacement vector with respect to the optic nerve head for each condition (Figure 3G). In healthy mice, the displacement of the ${\text{CX}}_{3}{\text{CR}}_{1}^{+/-}$ EGFP cells was not directional (Figures 3F,G), suggesting that cells are not preferentially moving toward or away from the optic nerve. In EAU, most of the motile cells moved centripetally, toward the optic nerve (Figures 3G,I), as revealed by the global displacement vector oriented toward the optic nerve head. This implies that a subset of ${\text{CX}}_{3}{\text{CR}}_{1}^{+}$ cells were activated within the retina and migrated through the optic nerve head, probably gaining access to inner parts of the CNS.

In order to determine the directionality of ${\text{CX}}_{3}{\text{CR}}_{1}^{+}$ cells relative to the optic nerve, we calculated the vectors defined by the optic nerve head and the starting and end point of the cell trajectory, respectively, for each motile ${\text{CX}}_{3}{\text{CR}}_{1}^{+}$ cell (Figure 3H). The vector sum of these two vectors projected on one dimension results in the displacement vector toward or away from the optic nerve (Figure 3H) for each cell.

### Longitudinal Calcium Imaging Reveals No Evident Signs of Calcium-Associated Neuronal Retinal Dysfunction during Onset and Peak of EAU

As previously demonstrated by us and others, sustained elevated calcium levels in neurons, i.e., 1 μM intracellular calcium over an hour, lead to strong morphological changes and ultimately to neuronal death, both in primary neuronal cultures and under *in vivo* conditions (22, 24). We have shown that enhanced contact of axons with peripheral immune cells in the brain stem of mice affected by EAE correlates with an increased calcium baseline in axons (22, 24, 27). Since the *TN L15* construct reacts slowly to calcium, over hundreds of milliseconds, it is able to specifically track only long-lasting increases of intracellular calcium. Thereby, it reports non-physiological, lasting elevations of the intracellular calcium concentration, but not the physiologic calcium oscillations within neurons. Under physiologic conditions, the construct records only the low average baseline (≈100 nM) (27). In CerTN L15 mice mainly the ganglion cells, which expressed Thy1 (28), encode the *TN L15* construct as we demonstrated by immunofluorescence analysis (Figure S3 in Supplementary Material).

**Figure 4:**
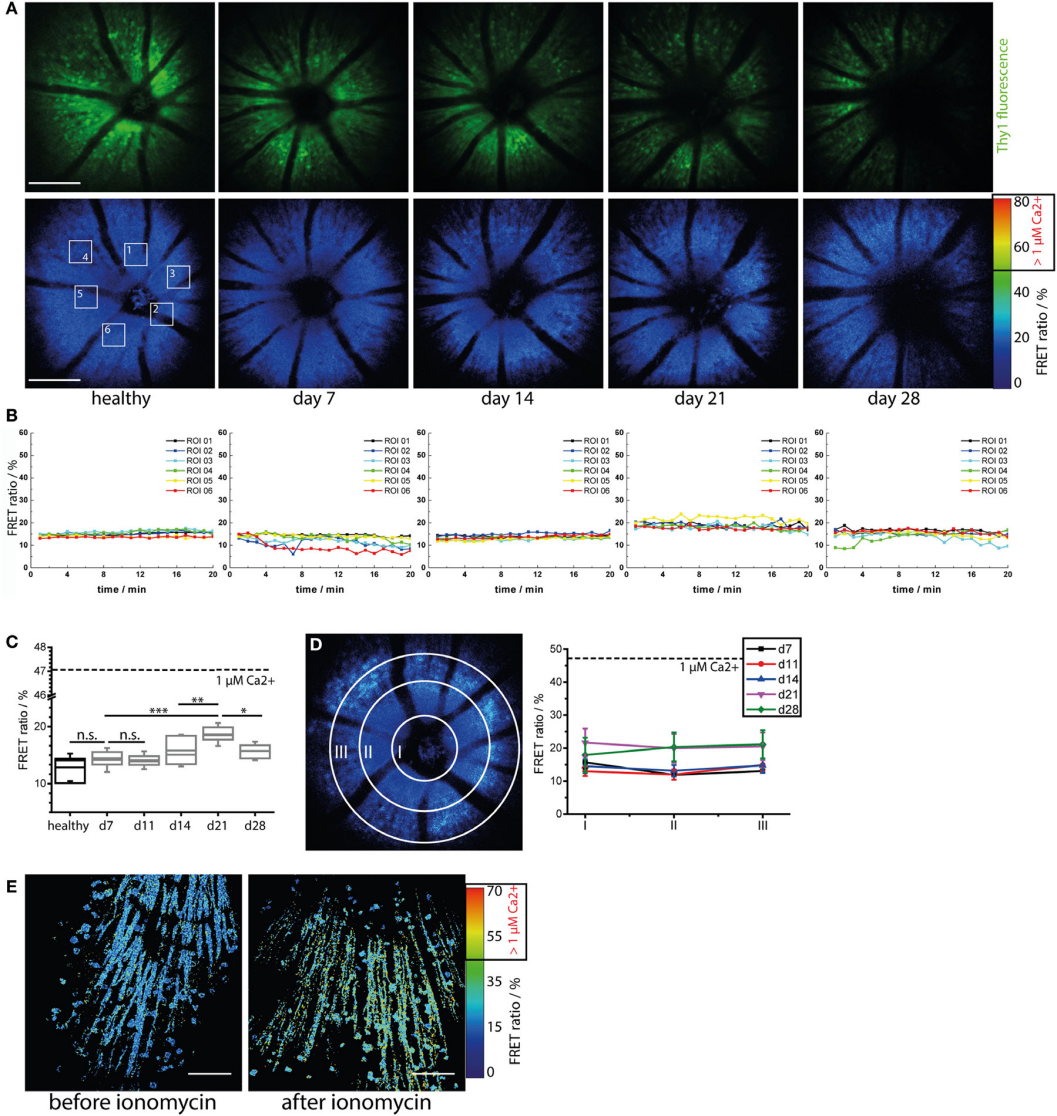
**Immune infiltration and microglial activation do not lead to calcium-associated neuronal dysfunction in the retina during onset and peak of experimental autoimmune uveoretinitis (EAU)**. **(A)** Representative three-dimensional projections of fluorescence retinal images (upper row) and FRET ratio images (lower row) of 1,400 μm × 1,400 μm × 300 μm in a healthy and in a *CerTN L15* mouse affected by EAU at time point days 7 (pre-onset), 14 (onset), 21 (peak), and 28 (peak) during the course of the disease. TN L15, a FRET-based Ca-sensor, is genetically encoded in neurons and expressed under the Thy1 promoter. The sensor contains Troponin C as Ca-sensitive protein and Cerulean and Citrine as FRET-donor and FRET-acceptor molecules, respectively. The FRET ratio, which scales with the intracellular calcium concentration, does not change during the course of EAU. λ_exc_ = 850 nm, λ_detection_ = 466 ± 30 nm (Cerulean), 525 ± 25 nm (Cerulean, Citrine), and 593 ± 20 (Citrine). *z*-step = 15 μm. Scale bar = 200 μm. **(B)** Time courses of the mean FRET ratio within the marked regions of interest (1–6) over 20 min for all time points depicted in **(A)**. No changes in the neuronal calcium was observed during the imaging time period, indicating that our two-photon microscopy of the eye did not disturb tissue function and is a reliable probe for identifying possible physiologic or pathologic changes. **(C)** Quantification of the mean FRET ratio of the whole imaged volume in the retina in healthy and in mice affected by EAU at different phases of the disease (*n* = 3 healthy mice, *n* = 4 EAU mice, two independent EAU experiments). The mean FRET ratio indicative for neuronal calcium increases transiently up to day 21 after immunization (peak EAU) but remains under the boundary of neuronal dysfunction (1 μM calcium, corresponding to 47% FRET ratio). **(D)** Adopting the same principle of concentric segmentation of the retina around the optic nerve head as in Figure 2 the mean FRET ratio in the Regions I–III was quantified for each measured time point after immunization, in all *CerTN L15* mice affected by EAU. In contrast to the immune infiltrate, which shows directed concentric spreading kinetics starting from the optic nerve head, the neuronal calcium shows an almost constant value all over the imaged retinal volume during both onset and peak EAU. **(E)** The retina of a healthy *CerTN L15* treated with ionomycine (40 μM) for 5 min shows increase of neuronal calcium above the threshold of neuronal dysfunction, i.e., 1 μM intracellular calcium corresponding to 47% FRET ratio. Statistical evaluation was determined using ANOVA one-way tests and Bonferroni *post hoc* test for multicolumn analysis (*p < 0.05, **p < 0.01, and ***p < 0.001).

Repeated neuronal calcium measurements in CerTN L15 mice affected by EAU revealed a significant increase of neuronal calcium in the GCL at day 21 after immunization as compared to the pre-onset phase (days 7 and 11 after immunization) and onset phase (day 14 after immunization), which faded at day 28 after immunization (Figures 4A–C; Videos S10–S12 in Supplementary Material). This result may reflect a sustained pathology of the retina, as previously shown in other organs of the CNS. However, despite strong immune infiltration with CD4^+^ and LysM^+^ cells, as well as alterations of the microglial network toward an activated morphology, the increase of neuronal calcium in the retina of EAU mice did not reach the typical intracellular calcium level of pathologic neuronal dysfunction found in the brain stem of mice affected by EAE under qualitatively similar immune infiltration conditions (24). Moreover, the neuronal calcium levels were similar in all cells within the concentric areas I–III around the optical nerve head and did not increase during the imaging period of 20 min (Figures 4B,D) at any stage of the disease. Hence, retinal inflammation at the onset and peak of EAU (up to day 28 after immunization) did not correlate temporally or spatially with neuronal tissue dysfunction related to calcium signaling and did not appear to primarily target the neuronal retina (GCL). We cannot exclude that neuronal dysfunction may develop independently of a disturbance of intracellular calcium, in which case our approach would fail. Furthermore, although we found in our experiments that the maximum neuronal calcium increase is reached at day 21 after immunization, repeated imaging experiments using our approach during the chronic phase of EAU (e.g., between days 28 and 60 after immunization) would shed light on possible delayed effects of inflammation on the neuronal retina.

As we did not observe any pathologic increase of neuronal calcium during the time-lapse imaging experiment (20 min, Figure 4B; Figure S2C in Supplementary Material), we conclude the retina of healthy and EAU mice are not functionally affected by exposure to the focused laser beam of the multiphoton microscope. Moreover, we were able to induce calcium-associated neuronal dysfunction and damage in the retina, i.e., FRET ratio of up to 70% corresponding to neuronal calcium of 3 μM, by local application of ionomycine 40 μM in healthy CerTN L15 mice (Figure 4E). Hence, our approach can reliably quantify calcium-associated functional state in retinal neurons. Furthermore, we confirmed in this way that distorted calcium signaling in retinal neurons during onset and peak of EAU is only a minor aspect of the disease.

## Discussion

As the only part of the CNS exposed to the outer environment, the eye is the ideal candidate for non-invasive intravital imaging of autoreactive attacks of the immune system on the CNS, typical for chronic neuroinflammatory pathologies. Independent of the specific CNS region being the direct target of the immune attack, the clinical involvement of the neuronal retina has been demonstrated in various contexts of neuroinflammation. Accordingly, the thinning of the GCL and of the retinal neuronal fiber layer are well described in MS and NMO and represent promising diagnostic approaches to evaluate the disease course (29). On the other hand, models of autoimmunity in the mouse eye are particularly relevant to understand the pathological mechanisms of neuroinflammation. They are necessary to evaluate the relation between brain and retinal inflammation and, thus, to take full advantage of the eye as a window to the CNS.

Multiphoton microscopy allows unique access to information on cellular dynamics within living organisms beyond the information on cellular composition and morphological tissue changes provided by static histology. Up to now, the technology has been widely used to elucidate basic physiologic processes as well as patho-mechanisms in various organs and disease models (30–32). In the context of neuroinflammation, which involves highly dynamic, tissue-dependent processes, the relevance of intravital multiphoton microscopy as a central investigation tool becomes obvious. Moreover, since neuroinflammatory processes are lasting days, weeks, or even months, whereas typical imaging time-windows in intravital microscopy are several hours, technologies allowing for repeated imaging in one and the same animal are requested. The potential of retinal two-photon imaging has been underlined by several recent publications (16, 18); however, to our knowledge, this is the first report demonstrating repeated time-lapse imaging of the retina, allowing us to monitor cellular dynamics, cellular interactions, and tissue function longitudinally in one mouse. Our approach reduces the number of animals needed for experiments and decreases inter-individual variance due to different responses to immunization across mice. Tissue and even cellular fate can be tracked over weeks and seemingly static processes are revealed as slow but dynamic.

Using our novel approach, we found dramatic differences in the motility behavior of effector cells—CD4^+^ T cells and phagocytes (LysM^+^ cells)—in the eye compared to the brain stem and spinal cord under comparable inflammatory conditions. In the brain stem and spinal cord, effector cells from the periphery moved directionally and rather rapidly (5–7 μm/min), especially along *de novo* built collagen fibers, similarly to their movement within secondary lymphoid organs (33). They enter the immune-privileged brain tissue *via* meninges or leaky blood vessels and accumulate at lesion sites (22, 33). In the eye, these cell subsets show a reduced displacement rate (approximately 1 μm/min). However, during the course of the disease (days to weeks), they move in a highly directed manner from the optic nerve head toward the retinal periphery. In line with this finding, we could not detect the collagen fibers characterized by second-harmonic generation at any time point, although they have previously been shown in imaging studies of the CNS (33) and of lymphoid organs to be used for directed cell movement.

Confirming previous findings, we observed that CD4^+^ T cells invaded the retina slightly earlier than LysM^+^ cells (phagocytes) and presumably initiate the inflammation. The immune infiltration typically began at the head of the optic nerve head, indicating cell migration from inner areas of the CNS through the optic nerve as a main pathway of retinal infiltration next to the immune infiltration through the disrupted blood–retina barrier. Interestingly, CD4^+^ T cells are significantly slower than LysM^+^ cells during the whole course of EAU. Considering the lack of collagen fibers in the inflamed retina, a possible explanation of this observation is that CD4^+^ T cells depend on the collagen fibers as highways facilitating their movement stronger than LysM^+^ cells do.

Taken together, our findings lead to the hypothesis that the CD4^+^ T cells, as initiators of the retinal inflammation, are locally reactivated directly at the optic nerve head and not within the perivascular space, as we do not see a compartmentalization to the retinal vasculature, unlike in the inflamed brain (26). Moreover, we hypothesize that LysM^+^ cells attracted to the retina by CD4 T cells deliberately search for tissue-specific targets for a direct attack, as they show invasive migration patterns similar to those in the brain.

Which CD4 T cells subsets, e.g., Th17 or Th-GMCSF cells, are relevant for the induction of autoimmunity in the eye, and how do they dynamically interact with the retina and with antigen presenting cells? What role do other cellular subsets, such as CD8 T cells and other soluble factors (e.g., CXCL12 or TNFα), play in this context? These are still open questions to be addressed in future studies.

In terms of pathological changes in the CNS compartments during neuroinflammation, a shift of the microglial network from a probing toward an activated morphology revealed the same local signs of gliosis previously described in the brain stem and spinal cord (34). In contrast to the results of intravital imaging studies of the inflamed brain (35, 36), phagocytic ${\text{CX}}_{3}{\text{CR}}_{1}^{+}$ cells (characterized by amoeboid shape) only moved rapidly in the perivascular space of the inflamed eye, probably because there were no conduits to orient their motion in the retinal parenchyma. Which factors inhibit or trigger the conduits for immune cells in the CNS is not yet known. Interestingly, during inflammation the ${\text{CX}}_{3}{\text{CR}}_{1}^{+}$ cells move perivascularly toward the optic nerve head, implying that cell immigration can be initiated in the eye during neuroinflammation and that the cells then move on *via* the optic nerve toward the inner regions of the CNS. This suggests that the fiber tracks of the optic nerve can act as a bidirectional highway for migrating cells.

Based on these observations, we assumed that in our model, not only the microglial network in the retina but also the neuronal compartment may be affected. Surprisingly, the GCL seems to be able to compensate damaging mechanisms associated with neuronal calcium in our model. Repeated neuronal calcium imaging, using a FRET-based calcium indicator genetically expressed under the Thy1 promoter in CerTN L15 mice (37), showed rather low and only transient dysfunctional calcium signaling in these neurons. In the inflamed brain, the highly dynamic peripheral immune inflammation and gliosis lead to neuronal dysfunction (34, 38), neuronal damage, and finally to neuronal death already at early stages of the disease. In contrast, the inflamed eye showed massive, slow but directed immune infiltration from the optic nerve head toward the retinal periphery, similar gliosis, but only negligible calcium-associated neuronal dysfunction at onset and peak of EAU. It should be noted that, due to the differential expression of the calcium sensor in our model, not all neuronal cells were visible, particularly rods and cones. A delayed effect of the inflammation on the calcium signaling of the retinal ganglion cells cannot be excluded. Repeated retinal intravital imaging experiments during the chronic phase of the disease will shed light on a possible accumulation of calcium-associated neuronal stress leading to late neuronal damage.

Moreover, the extent of neuronal damage to inner CNS compartments in our model, the EAU, is unclear. Similarly, reversible or irreversible effects of pathogenic CD4^+^ T cells, phagocytes (macrophages and activated microglia) and activated astrocytes on the neuronal function of the retina, in EAE, have not been investigated yet and are also unclear. If the latter is confirmed, the novel technology presented here will make non-invasive monitoring over time of treatment effects in chronic neuroinflammation and therapeutic tracing on single cell level in one individual possible. On the one hand, this will lead to a better understanding of relevant patho-mechanisms and, on the other hand, to a dramatic reduction of experimental animals.

The newly developed retinal imaging approach presented here promises to yield new insights not only into neuroinflammatory but also into neurodegenerative mechanisms. Retinal pathology has recently been intensively discussed in the context of Alzheimer disease, Parkinson disease, and amyotrophic lateral sclerosis (39, 40). Furthermore, unprecedented breakthroughs are possible in the context of pathologies such as genetic vasculature diseases and diabetes type I (41, 42), which are associated with changes of the retinal vasculature, including main blood vessels and capillaries. In terms of elucidation of immune responses, the combination of our approach with genetic EAU models (11), in which tertiary lymphoid organs have been detected within the retina, will allow monitoring of lymphocytic maturation over the whole duration of the immune reaction.

## Materials and Methods

### Two-Photon Laser-Scanning Microscopy (TPLSM)

Multiphoton fluorescence imaging experiments were performed using a specialized laser-scanning microscope based on a commercial scan head (TriMScope II, LaVision BioTec, Bielefeld, Germany). The detection of the fluorescence signals was accomplished with photomultiplier tubes in the ranges of 466 ± 20, 525 ± 25, and 593 ± 20 nm. Cerulean was excited at 850 nm and detected at 466 ± 30 nm simultaneously with citrine emission at 525 ± 25 and 593 ± 20 nm. Blood vessels were labeled with sulforhodamine 101 (excited at 900 nm and detected at 593 ± 20 nm) or FITC dextrane (excited at 800 nm and detected at 525 ± 25 nm). EGFP labeled microglia were excited at 900 nm and detected at 525 ± 50 nm. eYFP labeled CD4^+^ T cells were excited at 900 or 960 nm and detected at 525 ± 20 nm. tdRFP labeled LysM^+^ cells were excited at 1,100 nm and detected at 593 ± 20 nm. In all imaging experiments, an average maximum laser power of 50 mW was used to avoid photodamage. The acquisition time for an image with a field of view of 1,400 μm × 1,400 μm and a digital resolution of 994 pixel × 994 pixel was 1.6 s. To observe cell movement in different retinal layers, we acquired 300 μm large *z*-stacks each minute over a total time course of 20 min. Longer imaging sessions are possible with our setup; however, we limited the imaging time window to 20 min to avoid possible side effects due to mechanical stress or long-term laser damage on the retina.

### Data Analysis

As previously described, we defined the area of neuronal dysfunction as the area of free neuronal calcium exceeding a concentration of 1 μM. The neuronal calcium concentration was measured *in vivo* in mice expressing the FRET-based calcium biosensor TN L15 in Thy1^+^ cells. The FRET efficiency, which directly correlates with the intracellular calcium concentration, was measured ratiometrically as previously described by us (27). Briefly, the intensity *I* of the three acquisition channels 466, 525, and 593 were background corrected *I*_B_, smoothed with a Gaussian filter and corrected for spectral sensitivity η of the photomultiplier tubes. The 525 and 593 channels were additionally corrected for the spectral overlap α originating from Cerulean. The relative acceptor FRET signal FRET acceptor was calculated as follows:

$$\begin{array}{ll} {\text{FRET}}_{\text{acceptor}} & =\frac{I_{\text{YFP}}}{I_{\text{YFP}}+I_{\text{CFP}}}\\ \; & =\frac{\frac{\alpha_{525}}{\eta_{525}}\cdot \left(I_{525}-I_{\text{B},525}\right)+\frac{\alpha_{593}}{\eta_{593}}\cdot \left(I_{593}-I_{\text{B},593}\right)}{\frac{\alpha_{525}}{\eta_{525}}\cdot \left(I_{525}-I_{\text{B},525}\right)+\frac{\alpha_{593}}{\eta_{593}}\cdot \left(I_{593}-I_{\text{B},593}\right)+\frac{\alpha_{466}}{\eta_{466}}\cdot \left(I_{466}-I_{\text{B},466}\right)} \end{array}$$

Image segmentation and tracking of all cells were performed using existing segmentation, object-recognition and tracking plugins in FIJI, ImageJ. Statistical analysis of the data was performed using Graph Pad Prism.

### Mice

All mice used were on a C57/Bl6 background. The *CerTN L15* × *LysM tdRFP* mouse expresses a FRET-based calcium biosensor consisting of Cerulean (donor) and Citrine (acceptor) bound to troponin C, a calcium-sensitive protein present in certain subsets of neurons. Additionally, tdRFP was expressed in LysM^+^ cells. The $CX_{3}CR_{1}^{+/-}$ *EGFP* mouse was used to detect microglia and CD4^+^ eYFP mouse was used to detect effector T cells.

### EAU Induction

Experimental autoimmune uveoretinitis was induced as previously described. Briefly, mice were immunized subcutaneously with 200–300 μg of IRBP_1–20_ (AnaSpec, Belgium) emulsified in CFA (BD Difco, Germany) and were administered 200 ng pertussis toxin (List Biological Laboratories, Inc.) intraperitoneally at the time of immunization and 48 h later. Intravital multiphoton microscopy was performed at different stages of the disease, i.e., pre-onset, onset and peak (days 7, 11, 14, 21, and 28). According to the grading system of Xu et al. (23), we performed clinical scoring for lesions and infiltration in the optic disc, retinal vessels, and retinal tissue for both eyes (days 7, 14, 21, and 28).

### Surgical Preparation for Longitudinal Intravital Imaging

Animal experiments were approved by the appropriate state committees for animal welfare (G0093/15, LAGeSo—Landesamt für Gesundheit und Soziales) and were performed in accordance with current applicable guidelines and regulations. All TPLSM experiments were performed under deep isoflurane (1.5%) breathing anesthesia. Before each imaging session the pupil were widened with a drop of sterile eye drop solution (mixture of 2% phenylephrine and 0.4% tropicamide). The mounting of the mice to the positioning system and alignment of the retinas relative to the objective lens were performed in less than 10 min for each mouse, so that the required total anesthesia time was less than 30 min. A drop of sterile transparent eye salve (Vidisic, Bausch&Lomb) between cornea and objective lens were used to prevent eye dehydration. In some mice, the blood vessels were labeled by i.v. injection of 50 μl solution FITC dextrane or sulforhodamine 101.

### HE Histology

Eyes were harvested at day 28 after immunization, fixed in 4% paraformaldehyde, embedded in optimal cutting tissue, and frozen with liquid nitrogen. The 10-μm thick retinal cross sections were stained with standard hematoxylin and eosin. The severity of EAU was evaluated by a blinded operator based on the number and size of lesions and infiltrates. Nine mice were included in the histological examination.

### Retinal Flat Mounts

Whole retinas were surgically isolated from explanted, unfixed eyes as described previously (18), flattened on a glass slide, covered by medium, and finally sealed with a cover slip. For vasculature labeling, mice received an i.v. injection with labeled dextrane prior to sacrifice.

### Immunofluorescence

Cryosections were prepared for immunofluorescence staining as follows: sections were rehydrated in PBS and blocked with PBS containing 1% BSA, 1% Tween, and 10% FCS. Six retina cross sections per eye were used for each staining. Astrocytes were investigated with an Alexa 488 conjugated GFAP antibody. Activated microglia and macrophages were stained with Iba1 as the primary antibody followed by Alexa 647 as the secondary antibody. Cell nuclei were stained with DAPI. Between each staining step sections were washed with PBS solution containing 1% BSA and 1% Tween. Sections were embedded with DAKO fluorescent mounting medium.

### Fundoscopy

All fundoscopy experiments were performed directly before the TPLSM experiments under deep isoflurane (1.5%) breathing anesthesia. Pupils were dilated and kept moist as described above. Fundus images were acquired under a circular illuminated bright-field ocular equipped with a reflex camera (EOS600D Canon). To compensate the refractive power of the cornea air interface a thin cover slide was placed in front of the cornea. Fundus images were taken for both eyes in less than 5 min.

## Ethics Statement

Landesamt fuer Gesundheit und Soziales, Berlin, Germany. Animal experiment license G0093/15.

## Author Contributions

DB, HR, AH and RN designed and performed the research, analyzed the data, and wrote the manuscript. RN, HR, AB, FP, and AH initiated, organized, and supervised the project. HR and AH provided expertise in mouse handling and intravital imaging and performed EAE experiments (G0093/15). FP, RG, RL, RM, HZ, and AB performed experiments. RN, DB, JH, and VA developed the setup for longitudinal retinal time-lapse imaging.

## Conflict of Interest Statement

VA (LaVision Biotec) and JH (Luigs & Neumann) declare competing financial interests. The water-immersion objective lens will be commercialized by LaVision Biotec, Bielefeld, Germany and the positioning system for retina imaging will be commercialized by Luigs & Neumann, Rattingen. The remaining authors declare that the research was conducted in the absence of any commercial or financial relationships that could be construed as a potential conflict of interest.

## Supplementary Material

Figure S1 **Characterization of disease symptoms in the experimental autoimmune uveoretinitis**. **(A)** Fundoscopy of C57/Bl6-J mice healthy or affected by EAU induced by immunization with IRBP peptide (1–20) reveals differences in health and different phases of the disease. **(B)** Clinical score in EAU assessed by fundoscopy (*n* = 10 mice encompassing, three EAU experiments). The error bars represent SD. **(C)** HE histology analysis of the retina in healthy mice and mice affected by EAU, 28 days after immunization shows dramatic immune infiltration all through the retinal layers and in the vitreous body as well as disorganized, wave-shaped retinal layers. Scale bar = 200 μm. **(D)** Immunofluorescence analysis of retina in health and in EAU shows an increase of Iba1 signal indicative for microglial activation towards a phagocytic phenotype and disorganization of the GFAP (astrocytic) structures as well as enhancement within the GCL. Scale bar = 200 μm. The retinal layer abbreviations: RPE, retinal pigment epithelium; RC, layer of rods and cones; ONL, outer nuclear layer; OPL, outer plexiform layer; INL, inner nuclear layer; IPL, inner plexiform layer; GCL, ganglion cell layer.

Figure S2 **Mouse surgery and handling for repeated intravital imaging of the retina do not induce inflammation or evident neuronal retina damage (A) 3D fluorescence images of the retina of a healthy ${\text{CX}}_{3}{\text{CR}}_{1}^{+/-}$ EGFP mouse acquired at days 21, 25, 28, 35, and 42 after gluing the positioning cap on the skull**. The time points correspond to days 7, 11, 14, 21, and 28 after immunization in EAU experiments, since the immunization is always performed 2 weeks (14 days after gluing the positioning cap). **(B)** 3D fluorescence images of the retina of a healthy CD4.eYFP mouse acquired at days 21, 25, 28, 35, and 42 after gluing the positioning cap on the skull. Scale bar = 200 μm. **(C)** Results of time-lapse imaging (20 min) of the FRET signal within the retina of a healthy CerTN L15 × LysM tdRFP mouse showing no changes in the neuronal calcium after five consecutive imaging sessions (days 21, 25, 28, 35, and 42 after gluing the positioning cap on the skull). **(D)** 3D fluorescence image within the same mouse at day 42 (after the last imaging session) shows intact neuronal processes and somata as well as no signs of infiltration with LysM tdRFP cells (mostly phagocytes). Scale bar = 200 μm.

Figure S3 **TN L15 construct is expressed in the ganglion cells (GCL), in the retina of CerTN L15 mice**. Fluorescence image of the native, fixed retina (transversal section) of a healthy CerTN L15 mouse shows only in the GCL-specific citrine fluorescence (green). The other retinal layers as well as the eye lens are visible due to their autofluorescence (blue). Scale bar = 200 μm.

Video S1 ***In vivo* two-photon imaging of the vasculature of the mouse retina**. The 1,000 μm × 1,000 μm fluorescence images series of retinal blood vessels labeled by FITC dextrane acquired at tissue depths between 0 μm (retina surface) and 300 μm. *z*-step = 6 μm. The main blood vessels (veins and arteries) meet in the optic nerve head (ON head).

Video S2 **Longitudinal time-lapse intravital imaging of the retina of a CD4 EYFP mouse affected by experimental autoimmune uveoretinitis (EAU) 21 days after immunization with irpb peptide 1–20 (peak of disease)**. Three-dimensional 1,400 μm × 1,400 μm × 300 μm fluorescence images were acquired every minute, over 20 min in the retina of CD4 eYFP mice affected by EAU, at day 21. The CD4 T cells are depicted in green, whereas the autofluorescence of retinol especially from the retinal pigment epithelium is depicted in blue. *z*-step = 6 μm.

Video S3 **Longitudinal time-lapse intravital imaging of the retina of the same CD4 EYFP mouse affected by experimental autoimmune uveoretinitis (EAU) as in Video S2 in Supplementary Material, 28 days after immunization (peak of disease)**. Three-dimensional 1,400 μm × 1,400 μm × 300 μm fluorescence images were acquired every minute, over 20 min in the retina of CD4 eYFP mice affected by EAU, at day 28. The CD4 T cells are depicted in green, whereas the autofluorescence of the retinal pigment epithelium is depicted in blue. *z*-step = 6 μm.

Video S4 **Longitudinal time-lapse intravital imaging of the retina of a LYSM.TDRFP mouse affected by experimental autoimmune uveoretinitis (EAU), 21 days after immunization (peak of disease)**. Three-dimensional 1,400 μm × 1,400 μm × 300 μm fluorescence images were acquired every minute, over 20 min in the retina of LysM.tdRFP mice affected by EAU, at day 21. The LysM cells are depicted in red, whereas the autofluorescence of the retinal pigment epithelium is depicted in blue. *z*-step = 6 μm.

Video S5 **Longitudinal time-lapse intravital imaging of the retina of the same LYSM.TDRFP mouse affected by experimental autoimmune uveoretinitis (EAU), 28 days after immunization (peak of disease)**. Three-dimensional 1,400 μm × 1,400 μm × 300 μm fluorescence images were acquired every minute, over 20 min in the retina of LysM.tdRFP mice affected by EAU, at day 28. The LysM cells are depicted in red, whereas the autofluorescence of the retinal pigment epithelium is depicted in blue. *z*-step = 6 μm.

Video S6 **Longitudinal time-lapse intravital imaging of the retina of a ${\text{CX}}_{3}{\text{CR}}_{1}^{+/-}$ EGFP mouse affected by experimental autoimmune uveoretinitis (EAU) at day 7 after immunization with interphotoreceptor retinoid-binding protein PEPTIDE 1–20 (pre-onset of disease)**. Three-dimensional 1,400 μm × 1,400 μm × 300 μm fluorescence images were acquired every minute, over 20 min in the retina of ${\text{CX}}_{3}{\text{CR}}_{1}^{+/-}$ EGFP mice affected by EAU, at day 7, 11, 14, 21, and 28 after immunization. The ${\text{CX}}_{3}{\text{CR}}_{1}^{+/-}$ EGFP cells, microglia and macrophages, are depicted in green, whereas the vasculature labeled by rhodamine is depicted in red. *z*-step = 6 μm. The sessile cells with a high number of processes are resting microglia. Activated microglia and peripheral macrophages adopt an amoeboid morphology indicative for their shift towards an activated phenotype.

Video S7 **Longitudinal time-lapse intravital imaging of the retina of the same ${\text{CX}}_{3}{\text{CR}}_{1}^{+/-}$ EGFP mouse affected bY experimental autoimmune uveoretinitis (EAU) as in Video S4 in Supplementary Material, at day 14 after immunization (onset of disease)**. Three-dimensional 1,400 μm × 1,400 μm × 300 μm fluorescence images were acquired every minute, over 20 min in the retina of ${\text{CX}}_{3}{\text{CR}}_{1}^{+/-}$ EGFP mice affected by EAU. The ${\text{CX}}_{3}{\text{CR}}_{1}^{+/-}$ EGFP cells, microglia and macrophages, are depicted in green, whereas the vasculature labeled by rhodamine is depicted in red. *z*-step = 6 μm.

Video S8 **Longitudinal time-lapse intravital imaging of the retina of the same ${\text{CX}}_{3}{\text{CR}}_{1}^{+/-}$ EGFP mouse affected bY experimental autoimmune uveoretinitis (EAU) as in Videos S4 and S5 in Supplementary Material, at day 21 after immunization (peak of disease)**. Three-dimensional 1,400 μm × 1,400 μm × 300 μm fluorescence images were acquired every minute, over 20 min in the retina of ${\text{CX}}_{3}{\text{CR}}_{1}^{+/-}$ EGFP mice affected by EAU. The ${\text{CX}}_{3}{\text{CR}}_{1}^{+/-}$ EGFP cells, microglia and macrophages, are depicted in green, whereas the vasculature labeled by rhodamine is depicted in red. *z*-step = 6 μm.

Video S9 **Longitudinal time-lapse intravital imaging of the retina of the same ${\text{CX}}_{3}{\text{CR}}_{1}^{+/-}$ EGFP mouse affected by experimental autoimmune uveoretinitis (EAU) as in Videos S4–S6 in Supplementary Material, at day 28 after immunization (peak of disease)**. Three-dimensional 1,400 μm × 1,400 μm × 300 μm fluorescence images were acquired every minute, over 20 min in the retina of ${\text{CX}}_{3}{\text{CR}}_{1}^{+/-}$ EGFP mice affected by EAU. The ${\text{CX}}_{3}{\text{CR}}_{1}^{+/-}$ EGFP cells, microglia, and macrophages, are depicted in green, whereas the vasculature labeled by rhodamine is depicted in red. *z*-step = 6 μm.

Video S10 **Time-lapse intravital imaging of the retina of a healthy CERTN L15 mouse reveals neuronal calcium and, hence, neuronal function is not disturbed by the laser beam during multiphoton microscopy**. Three-dimensional 1,400 μm × 1,400 μm × 300 μm FRET ratio images were acquired every minute, over 20 min in the retina of healthy CerTN L15 mice. In this mouse strain, some neuronal subsets express the TN L15 genetically encoded Ca^2+^ biosensor. The TN L15 biosensor is a FRET-biosensor based on Troponin C, a calcium-sensitive protein. The FRET pair contains Cerulean (cyan fluorescence) as donor and Citrine (yellow fluorescence) as acceptor. We used a three channel-based ratiometric FRET evaluation method to calculate the FRET ratio in the neurons and to evaluate the sustained neuronal calcium concentration. *z*-step = 6 μm.

Video S11 **Time-lapse intravital imaging of the retina of a CERTN L15 mouse affected by experimental autoimmune uveoretinitis (EAU), 21 days after immunization with interphotoreceptor retinoid-binding protein peptide 1–20 (peak of disease)**. Three-dimensional 1,400 μm × 1,400 μm × 300 μm FRET ratio images were acquired every minute, over 20 min in the retina of CerTN L15 mice affected by EAU at days 7, 11, 14, 21, and 28 after immunization. *z*-step = 6 μm.

Video S12 **Time-lapse intravital imaging of the retina of the same CERTN L15 mouse affected by experimental autoimmune uveoretinitis (EAU) as in Video S9 in Supplementary Material, at day 28 after immunization (peak of disease)**. Three-dimensional 1,400 μm × 1,400 μm × 300 μm FRET ratio images were acquired every minute, over 20 min in the retina of CerTN L15 mice affected by EAU. z-step = 6 μm.
