# Supplementary figures and images for: Longitudinal Intravital Imaging of the Retina Reveals Long-term Dynamics of Immune Infiltration and Its Effects on the Glial Network in Experimental Autoimmune Uveoretinitis, without Evident Signs of Neuronal Dysfunction in the Ganglion Cell Layer
Source: Front Immunol. 2016 Dec 23;7:642. doi: 10.3389/fimmu.2016.00642 (PMC5179567; doi:10.3389/fimmu.2016.00642)

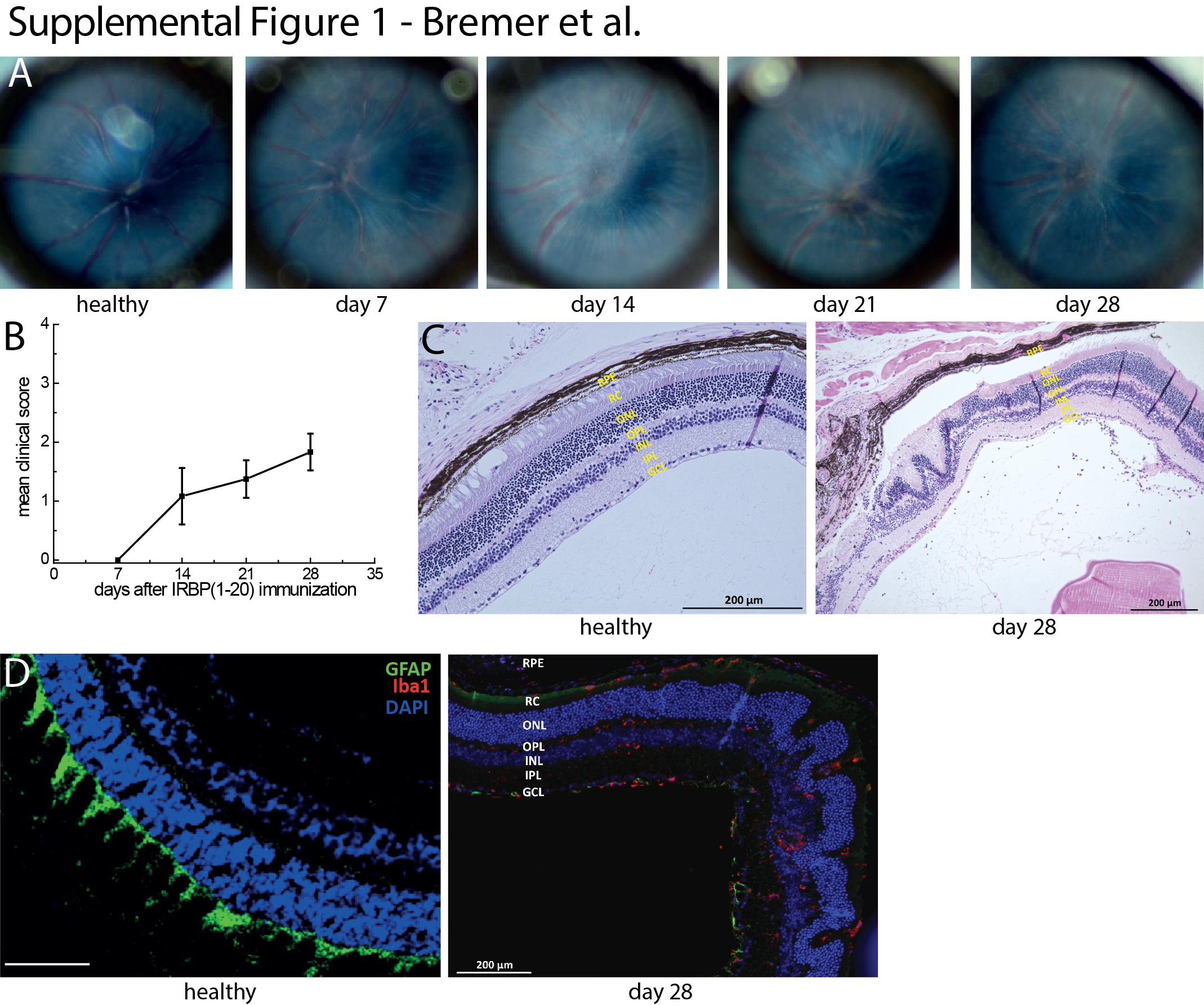

Supplement: Figure S1 — Characterization of disease symptoms in the experimental autoimmune uveoretinitis. (A) Fundoscopy of C57/Bl6-J mice healthy or affected by EAU induced by immunization with IRBP peptide (1–20) reveals differences in health and different phases of the disease. (B) Clinical score in EAU assessed by fundoscopy (n = 10 mice encompassing, three EAU experiments). The error bars represent SD. (C) HE histology analysis of the retina in healthy mice and mice affected by EAU, 28 days after immunization shows dramatic immune infiltration all through the retinal layers and in the vitreous body as well as disorganized, wave-shaped retinal layers. Scale bar = 200 μm. (D) Immunofluorescence analysis of retina in health and in EAU shows an increase of Iba1 signal indicative for microglial activation towards a phagocytic phenotype and disorganization of the GFAP (astrocytic) structures as well as enhancement within the GCL. Scale bar = 200 μm. The retinal layer abbreviations: RPE, retinal pigment epithelium; RC, layer of rods and cones; ONL, outer nuclear layer; OPL, outer plexiform layer; INL, inner nuclear layer; IPL, inner plexiform layer; GCL, ganglion cell layer. [file Image_1.PNG]

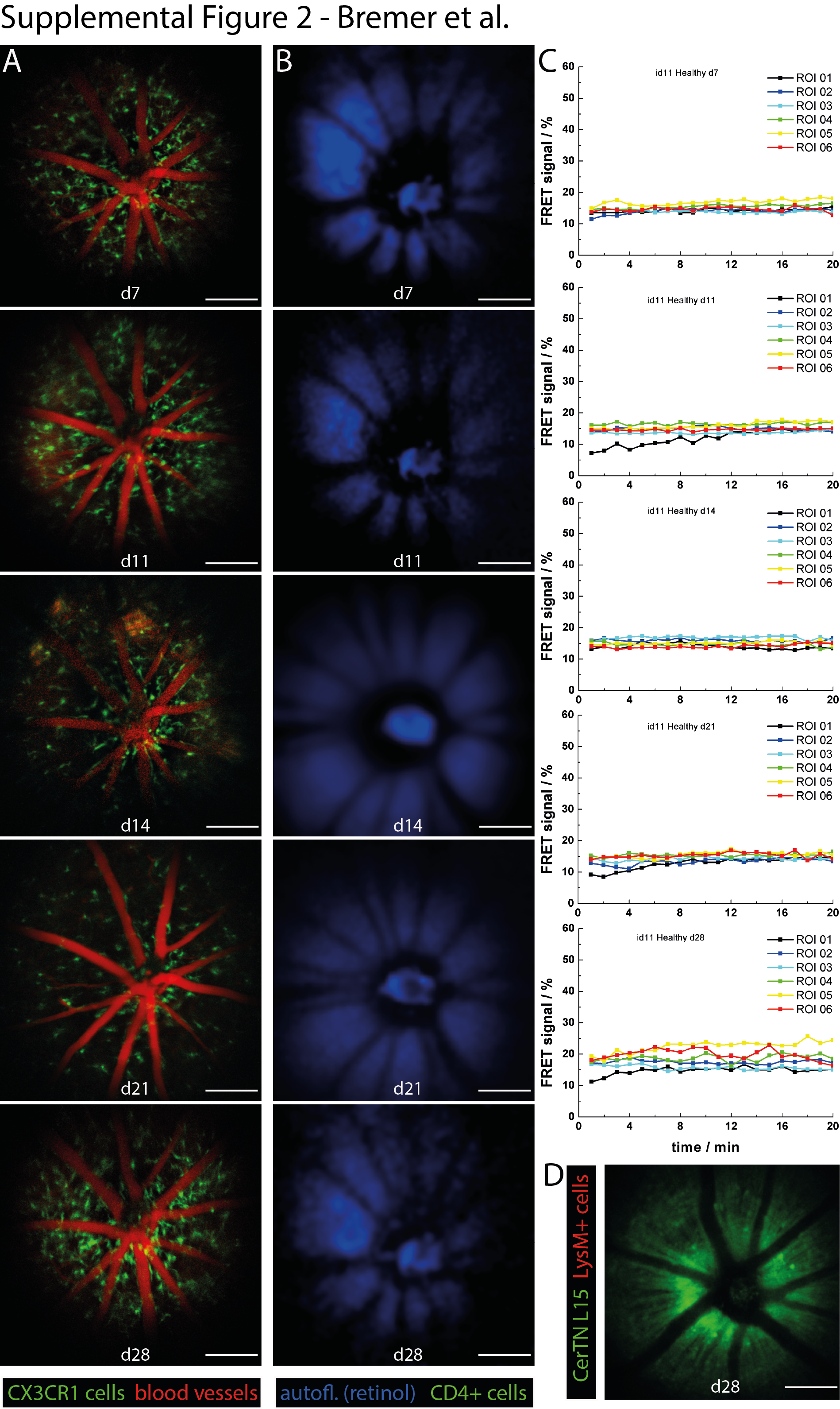

Supplement: Figure S2 — Mouse surgery and handling for repeated intravital imaging of the retina do not induce inflammation or evident neuronal retina damage (A) 3D fluorescence images of the retina of a healthy CX3CR1+/− EGFP mouse acquired at days 21, 25, 28, 35, and 42 after gluing the positioning cap on the skull. The time points correspond to days 7, 11, 14, 21, and 28 after immunization in EAU experiments, since the immunization is always performed 2 weeks (14 days after gluing the positioning cap). (B) 3D fluorescence images of the retina of a healthy CD4.eYFP mouse acquired at days 21, 25, 28, 35, and 42 after gluing the positioning cap on the skull. Scale bar = 200 μm. (C) Results of time-lapse imaging (20 min) of the FRET signal within the retina of a healthy CerTN L15 × LysM tdRFP mouse showing no changes in the neuronal calcium after five consecutive imaging sessions (days 21, 25, 28, 35, and 42 after gluing the positioning cap on the skull). (D) 3D fluorescence image within the same mouse at day 42 (after the last imaging session) shows intact neuronal processes and somata as well as no signs of infiltration with LysM tdRFP cells (mostly phagocytes). Scale bar = 200 μm. [file Image_2.PNG]

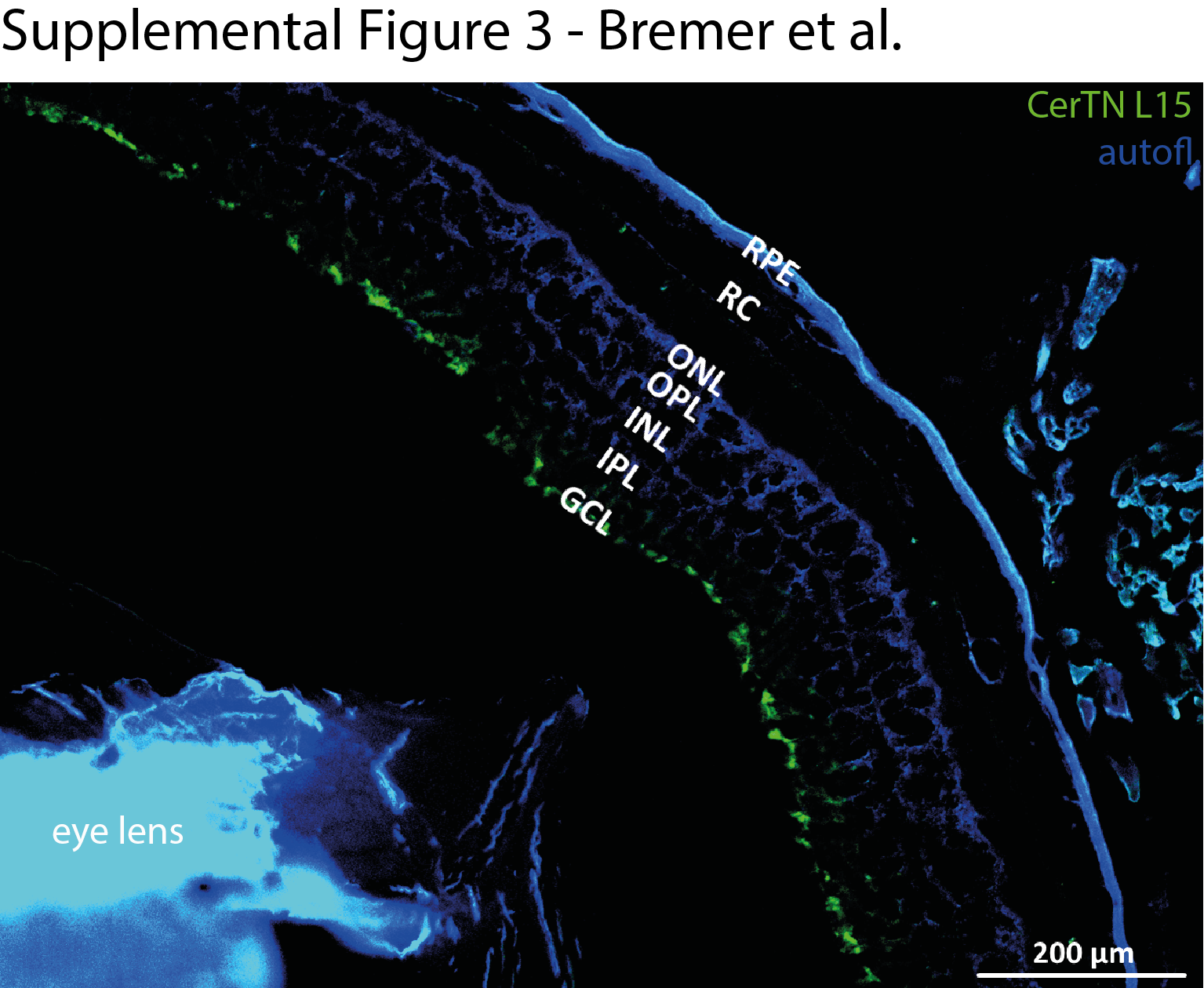

Supplement: Figure S3 — TN L15 construct is expressed in the ganglion cells (GCL), in the retina of CerTN L15 mice. Fluorescence image of the native, fixed retina (transversal section) of a healthy CerTN L15 mouse shows only in the GCL-specific citrine fluorescence (green). The other retinal layers as well as the eye lens are visible due to their autofluorescence (blue). Scale bar = 200 μm. [file Image_3.PNG]
